# Perimetric Inflammations

**Published:** 1879-11

**Authors:** H. P. Merriman

**Affiliations:** Professor of Medical Jurisprudence and Hygiene, Chicago Medical College


ARTICLE II.

PERIMETRIC INFLAMMATIONS. By H. P. MERRIMAN, M.D., Professor of Medical Jurisprudence and Hygiene, Chicago Medical College. Read before the Chicago Gynæcological Society, Sept. 26, 1879.

As this is the night for discussion, and not for the reading of a paper, I am glad it has fallen my turn to give direction to the thoughts of the society for the evening.

By perimetric inflammations I intend the inflammations immediately around the uterus, and mean all of them, whether affecting the peritoneum alone or the cellular tissue alone, or both of them together.

These inflammations have long been known to the profession under the names of pelvic abscess, iliac abscess, peri-uterine phlegmon, peri-metritis, pelvic cellulitis and inflammation of the uterine appendages.

The probability is, however, that only the severer forms were recognized — those attended with a high fever and usually terminating in suppuration. Such are the cases described by Doherty, McClintock and Hardy, Churchill, West, Simpson, Madam Boivin, Nonat Aran, Bernutz, Goupil, and others.

Most of these authors seem to have expected full and complete recovery sooner or later, and to have regarded the affection as important chiefly from the severity of the suffering accompanying it.

Even Thomas says he thinks there is no chronic form of "peri-uterine cellulitis;" that either resolution takes place in two or three weeks, or the affection goes on to suppuration, which he calls a result rather than a stage of the affection.

This acute form, though important and not rare, is still not very common. Were this the only way in which the affection shows itself, I should not have chosen it for our evening's consideration.

I think it cannot fail to have struck the attention of every physician who has practiced much in the department of women's diseases, that in making vaginal examinations he frequently finds evidence of previous perimetric inflammation.

I take the liberty of mentioning some of the most common evidences:

(*a.*) The uterus immovable and surrounded by adhesions.

(*b.*) The uterus anteflexed or retroflexed and fundus adherent, but cervix movable.

(*c.*) The uterus drawn to one side, but susceptible of some limited motion, though usually with pain.

*d.*) The tissue felt through the wall of the vagina, thickened and tense in some places, often as hard as a piece of sole leather; this is very observable when felt through the roof.

(*e.*) Tenderness on pressure in all these hard places.

(*f.*) Some places very tender to deep pressure, where there is no appreciable hardening.

(*g.*) Patient complains of pain in the iliac regions, especially when fatigued.

These symptoms appear to indicate that a more or less severe inflammation has occurred, with an effusion of coagulable lymph which has not been fully absorbed, and with remains still of the inflammation. In other words — with all deference to Dr. Thomas — there does exist a chronic pelvic cellulitis.

The seriousness of this complication is well recognized, as to—

The suffering it produces;

The danger of new activity ; and

The reaction upon other uterine ailments.

It is this chronic form of the affection which I hope will be thoroughly discussed to-night. The subject is toc broad for one evening, so I shall take up but a part of it.

As to the parts involved in these inflammations, a few cases have occurred and been substantiated by autopsy where only the cellular tissue near the uterus was involved; likewise a few where only the peritoneum was affected; but I believe observers agree that the clinical symptoms are not different whether one or more than one tissue is affected. The affection may start in the peritoneum, but it usually quickly extends to the cellular tissue in contact with it, and if it starts in the cellular tissue, it cannot become very general without involving the peritoneum.

For this reason I have used the term perimetric inflammation as being more general in its signification than any of the other names. The fact that the scholarly Dr. Barnes uses it, gives it authority also, but the term cellulitis has come into such general use to signify all these inflammations that it may be well still to use it, although pathologically incorrect.

*Location.* — As to the location of these attacks, Dr. Thomas says: "The usual, indeed the almost invariable seat of peri-uterine cellulitis is the areolar tissue of the broad ligaments, and generally that of one side only is affected."

Dr. West says: "The cellular tissue anywhere in the neighborhood of the womb may be the seat of the mischief, but that between the folds of the broad ligament is attacked far more often than the same structure in any other situation;" and he gives a table showing the location in 52 cases: in 34 of these it was in the broad ligament, and of this number the inflammation was on the left side in 25; in four of the 25, however, both sides were affected.

In 14 cases tabled by Dr. Thomas, we find the left broad ligament affected 12 times, but three of the 12 had also the right side affected.

Dr. Emmet, who has studied this affection more than any one else, gives a table of 157 cases of uncomplicated cellulitis occurring in his own practice, and in these the left broad ligament was affected 65 times and the right 16 times.

Other observers give about the same proportions, namely, that between 40 and 50 per cent. occur in the left broad ligament; general cellulitis occurs next in frequency, then inflammation behind the uterus, lastly inflammation on the right side and pelvic abscess, which were of the same frequency.

This remarkable frequency of cellulitis on the left side is explained by Dr. Jacobi as perhaps due to the superior development of the left cerebral hemisphere, it having a better and more direct supply of blood, which it receives by the anatomical arrangement of the large vessels springing from the left side of the aortic arch, which increased arterialization renders it more fit to innervate the right half of the body than is the case with the right cerebral hemisphere. The left side of the body being less perfectly innervated, is therefore also more liable to pathological changes.

The question of manner of origin in this affection is one of great importance, and deserves our special attention. There are but two theories at the present day which have followers :

One, that the disease is usually a secondary one, arising from prior inflammations in the ovaries, Fallopian tubes or uterus ; the other, that the disease, except when puerperal, is more frequently primary, spreading to the ovaries and Fallopian tubes from the cellular tissue.

The upholders of the former theory are numerous, and comprise nearly all the prominent names in modern gynæcology. Of the latter theory, Dr. Emmet is the prominent advocate. Although he stands thus almost alone, he has done so much for gynæcology, and is such a careful and conscientious observer, that his opinions are entitled to respect and proper consideration. The views he holds, if established, will revolutionize the theory and practice of gynæcology. Permit me to give them in his own language. After excepting the lesions of the puerperal state, he thus expresses his convictions :

" While the primary cause of disease lies through the influences of the sympathetic system in impaired nutrition, we must look to pathological changes in the connective tissue as the cause of the results we now regard as the original disease in the uterus and ovaries.

" I have never seen a case of ovaritis without inflammation of the neighboring tissues, and where I have had the opportunity of observing one early enough, I have always detected the cellulitis before the ovary became involved.

" We have no means of judging with any accuracy as to the condition of the Fallopian tubes during life. But the probabilities are, unless poisoned by gonorrhœa, that inflammation of its mucous membrane with that of the uterus, is secondary to some previous lesion in the cellular tissue."

In other words, Dr. Emmet believes that inflammations of the ovaries, Fallopian tubes and mucous membrane of the uterus do not occur primarily except as a consequence of the puerperal condition, of gonorrhœa, or of the use of instruments, as the uterine sound; but that it occurs secondarily, as a result of existing cellulitis. This he believes to be the rule rather than the exception.

With these views of Dr. Emmet I cannot agree. I may be pardoned for mentioning my own conclusions from comparatively limited experience. I find very frequently cases of endometritis where I can detect no trace of cellulitis by the most careful examination. Some of these cases have an abundant discharge from the uterus, with very little tenderness in its cavity; others have little discharge, but so much tenderness that the most gentle introduction of the probe gives exquisite pain, and the menstrual period of many of these is full of suffering; yet bimanual examination produces no pain in any part of the pelvis.

To three or four farther arguments which Dr. Emmet brings forward to support his views, I desire to call your attention.

He is inclined to believe that phlebitis is an important element in the causation of the affection we are discussing. Does not that make cellulitis a secondary or *even a tertiary* disease? For what is the cause of the phlebitis?

Dr. Emmet suggests the existence of a low grade of phthisis in the cellular tissue as perhaps the predisposing cause of idiopathic cellulitis. I do not know what he means by "a low grade of phthisis," as applied to the condition of the cellular tissue. If he means that the tissue is not well nourished, I understand him — otherwise not.

Dr. Emmet mentions, to support his views, the very tortuous course taken by the vessels and nerves in passing through the connective tissue to reach the uterus; they are so doubled upon themselves "that they cannot be put on the stretch or their calibre lessened by the traction from pregnancy or uterine displacement." This very fact shows to my mind that the cellular tissue in this locality is unusually protected, and has no more reason to become inflamed idiopathically than the same tissue in the popliteal space.

The pelvic connective tissue in the woman, it is true, is more liable to disease than in the male, because surrounding and in contact with organs that are subject to greater variations in blood and nervous supply, but not because these vessels and nerves pass *through* the connective tissue on their way to the ovaries and uterus. For these reasons I should expect disease of the connective tissue to be secondary.

I cannot see why idiopathic or primary cellulitis should arise any more frequently in a woman than in a man. Cellulitis does occur in the male sometimes, but it always follows inflammation either of the prostate, of the urethra, or of the rectum, or some surgical injury. In other words it is either by continuity or by reflex action. I have yet to learn of any idiopathic cases.

Cellulitis has occurred both before and after the child-bearing period, but all authors agree that this event is rare. Dr. Emmet accounts for it by saying that after the menopause there is a diminished supply of blood to the uterus and ovaries. This explanation does not seem sufficient, according to his theory, for although the supply *through* the connective tissue may be diminished, yet the supply *to* it is kept up as well as formerly.

After careful consideration of the whole subject, the conclusions of Dr. Matthews Duncan impress themselves upon my mind with renewed force. In his most excellent work upon perimetritis and parametritis he says:

" The cellular tissue and the peritoneal membranes are protected and are believed to be specially disinclined to original or idiopathic inflammatory action. Why then are they so frequently inflamed in this region?

" The theory on which I insist is that these inflammations are all secondary; that they are produced by inflammations of the uterus or of the Fallopian tubes or of the ovaries, or by noxious discharges through or from the Fallopian tubes or ovaries, or by mechanical injury. Without one or other of these causes the inflammation and abscess is not observed.

"Of all the prolific causes, inflammation of the mucous membrane of the womb is, in my opinion, the most common, and this both in the puerperal and non-puerperal states."

What, gentlemen, are your opinions upon this subject?

Again, what shall our treatment be for this condition? Dr. Emmet's suggestions seem to me the very best that can be followed. They are:

Hot water injections twice a day.

Good, nutritious food.

Regulation of bowels.

Sunlight and fresh air.

A great deal of rest, especially if there is much tenderness.

Suitable support to the pelvic contents.

Tonics and alteratives.

The judicious use of these, combined with proper kinds of exercise when tenderness is diminished, will help most cases.

